# A Biochemical Approach to Understand the Pathogenesis of Advanced Pulmonary Arterial Hypertension: Metabolomic Profiles of Arginine, Sphingosine-1-Phosphate, and Heme of Human Lung

**DOI:** 10.1371/journal.pone.0134958

**Published:** 2015-08-28

**Authors:** Yidan D. Zhao, Lei Chu, Kathleen Lin, Elise Granton, Li Yin, Jenny Peng, Michael Hsin, Licun Wu, Amy Yu, Thomas Waddell, Shaf Keshavjee, John Granton, Marc de Perrot

**Affiliations:** 1 Latner Thoracic Surgery Research Laboratories and Division of Thoracic Surgery, University Health Network, University of Toronto, Toronto, Ontario, Canada; 2 Clinical Studies Resource Centre, Toronto General Hospital, University Health Network, University of Toronto, Toronto, Ontario, Canada; Chinese Academy of Sciences, CHINA

## Abstract

Pulmonary arterial hypertension (PAH) is a vascular disease characterized by persistent precapillary pulmonary hypertension (PH), leading to progressive right heart failure and premature death. The pathological mechanisms underlying this condition remain elusive. Analysis of global metabolomics from lung tissue of patients with PAH (n = 8) and control lung tissue (n = 8) leads to a better understanding of disease progression. Using a combination of high-throughput liquid-and-gas-chromatography-based mass spectrometry, we showed unbiased metabolomic profiles of disrupted arginine pathways with increased Nitric oxide (NO) and decreased arginine. Our results also showed specific metabolic pathways and genetic profiles with increased Sphingosine-1-phosphate (S1P) metabolites as well as increased Heme metabolites with altered oxidative pathways in the advanced stage of the human PAH lung. The results suggest that PAH has specific metabolic pathways contributing to the vascular remodeling in severe pulmonary hypertension. Profiling metabolomic alterations of the PAH lung has provided a new understanding of the pathogenic mechanisms of PAH, which benefits therapeutic targeting to specific metabolic pathways involved in the progression of PAH.

## Introduction

Pulmonary arterial hypertension (PAH), a vascular disease characterized by persistent pulmonary hypertension (PH) could lead to progressive right heart failure and premature death [1]. Recent evidence shows that abnormal metabolic pathways may play a significant role in the development and progression of PAH [2]. A similar metabolic change has been identified as a feature of malignant tumor transformation, displaying hyperproliferative characteristics similar to pulmonary arterial endothelial cells (PAECs) in PAH [3,4]. Because mitochondrial oxidative phosphorylation (with glucose uptake and utilization) has been shown to occur in the pulmonary arterial endothelium of PAH patients [5], metabolic alterations in PAECs are more likely to be representative of disease development. Increased hemoglobin levels have been found in the PAH sample group with/without history of diabetes or any other obvious metabolic diseases, indicating the impairment of whole-body glucose homeostasis in PAH [6–8]. In animal models with chronic hypoxia-induced PAH, vascular changes characteristic of the disease have been directly linked to an imbalance between glycolysis, glucose oxidation, and fatty acid oxidation [9]. In addition, *in vitro* PA endothelial cell cultures with disrupted BMPRII gene also showed significant metabolomic changes [2].

Our recent work showed that disrupted glycolysis, increased TCA cycle, and increased fatty acid metabolites with altered oxidation pathways are characteristic of abnormal metabolism in the late stage of human PAH in lungs [4]. This indicates that PAH has its own specific metabolic pathways contributing to the abnormal ATP synthesis for the vascular remodeling process in pulmonary hypertension. Moreover, we provided direct evidence that *de novo* synthesis of bile acids in PAH lung tissue is associated with specific expression of the metabolic enzyme, CYP7B1, which may contribute to the vascular pathogenesis of PAH [4]. Collectively, *in vitro* human and animal models suggest that multiple metabolic pathways are reprogrammed during PAH vascular remodeling and that metabolic heterogeneity may play an important role in supplying ATP and contributing towards the molecular pathogenesis of PH. Here, we provide direct evidence that multiple metabolic heterogeneity exists in the human PAH lung. Our results show specific metabolic pathways and genetic profiles with increased Sphingosine-1-phosphate (S1P) metabolites, disrupted arginine pathways, and increased Heme metabolites with altered oxidative pathways in the later stage of the human PAH lung. These identified metabolites may serve as possible biomarkers for the diagnosis and prognosis of PAH. By profiling these metabolomic changes of the PAH lung, we revealed the scope of metabolic heterogeneity of PAH. As well, we established new pathogenic mechanisms of severe PAH, opening an avenue of exploration for therapeutics that target metabolic pathway alterations in the progression of PAH.

## Materials and Methods

### Patient data

The lung tissue of 8 PAH patients were obtained from end stage PAH patients that had undergone lung transplantation. Eight control lung samples were obtained from normal tissue of cancer patients undergoing lobectomy (Table 1) [4]. All patients provided written informed consent, in accordance with the Declaration of Helsinki, for research protocols approved by the University Health Network (UHN) Research Board (#110932_AE). The lung samples were from the bottom left lung and were collected and washed with PBS to remove the blood. The samples were quickly snap-frozen in the operating room and stored in −80°C prior to sample analysis. The PAH patient cohort included patients who were diagnosed based on WHO group l classification of PH according to the fifth World Symposium on Pulmonary Hypertension [10].

**Table 1:** Patient characteristics.

Sex	Diagnosis	age	sPAP	dPAP	mPAP	WP	CO	PVR	MvO2
Male	IPAH	57	89	20	55	12	3.2	1075	54
Female	Eisenmenger's	49	125	58	76	4	2.7	2133	67
Female	SLE,PAH	30	76	38	51	3	1.7	2259	39
Female	IPAH	35	91	14	55	1	3.4	1271	62
Female	IPAH	47	80	30	47	0	3.4	1106	74
Female	SLE,PAH	38	92	45	61	5	5.2	845	53
Female	CHD PAH	19	75	34	50	9	4.6	713	65
Female	IPAH/SLRx	50	77	29	51	12	2.1	1486	
	Mean	41	88	34	56	6	3	1361	59
	STDEV	12	16	14	9	5	1	568	12

Notes: IPH: Idiopathic pulmonary arterial hypertension; SLE: systemic lupus erythematosus; CHD: congenital heart disease; sPAP: systolic pulmonary artery pressure; mPAP: Mean pulmonary artery pressure; MvO2:Mixed venous oxygen saturation

### Metabolomic profiling

Metabolomic analysis was carried out according to the procedure previously described [4]. In short, the samples were processed using the automated MicroLab STAR system (Hamilton Co, Reno, NV, USA). Using aqueous methanol extraction, samples were prepared by removing the protein fraction while allowing maximum recovery of small molecules. Four fractions of the extraction were used for metabolomics performance: the first for analysis by UPLC/MS/MS (positive mode), the second for UPLC/MS/MS (negative mode), the third for GC/MS, and the last for backup. Using the appropriate instrument (either UPLC/MS/MS or GC/MS), samples were then prepared [4]. To identify biochemicals that were significantly different between experimental groups, a Welch’s two-sample t-test was performed-following log transformation and imputation with minimum observed values for each compound. Data analysis was completed based on statistical significance (as well as those approaching significance (0.05<p<0.10). Each metabolite was assigned a pathway in order to examine the impact of an increased or decreased metabolite on the overall pathway mechanism.

### Transcriptomic analysis

Idiopathic PAH lung tissue and human control lung tissue were used for global profiles. The analyses for total RNA in lung tissue were performed as formerly outlined [4]. Shortly, biotinylated cRNA was prepared from 6 ug total RNA (Expression Analysis). 10 ug of cRNA was hybridized at 45C for a time period of 16 hours on GeneChip Genome Array. The data were determined with Partek Genomics Suite 6.6 using Affymetrix default analysis settings and global scaling as the normalization method. Using Partek Genomics Suite 6.6, the value definition was set up and using a t-test with a false discovery rate (FDR) of 2 fold, P<0.01 was determined. The database has been submitted to NCBI/GEO and has been approved and assigned a GEO accession number, GSE53408.

## Results

PAH lung samples displayed broad changes in arginine and sphingolipid metabolism. Significant changes were also observed in the heme metabolism compared to control lungs. We additionally performed transcriptomic analyses for genes encoding enzymes that regulate the affected metabolic pathways.

### PAH lungs showed a significant alteration of multiple interdependent metabolic pathways compared with control lungs

**Fig 1:**
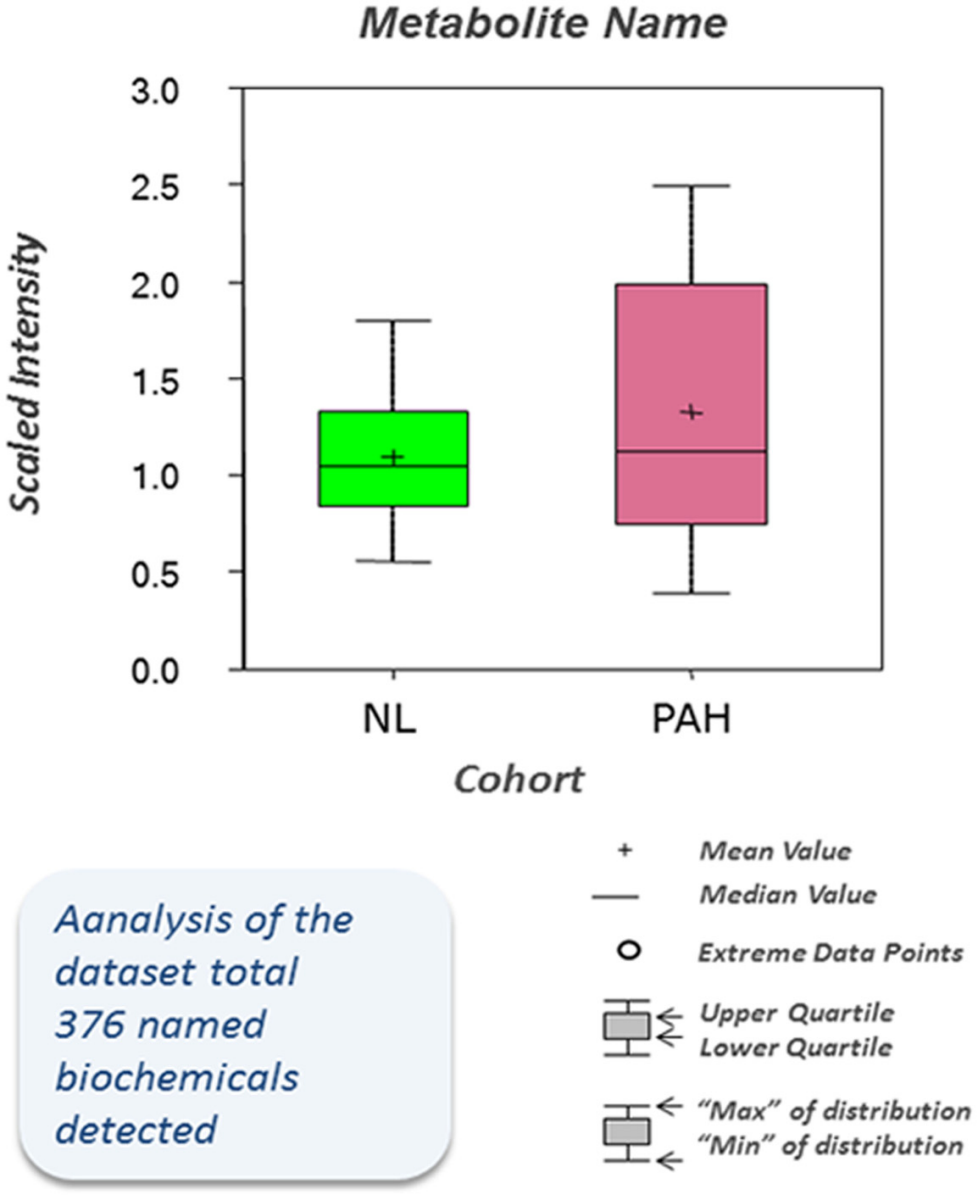
Metabolomic profiling of pulmonary hypertension. Analysis of the dataset total 376 named biochemical was detected. Statistical comparisons using Welch's Two-Sample t-Test show significantly altered biochemicals in PAH samples (N = 8) compared with biochemical profiling in normal samples (N = 8). The biochemical profiles of PAH tissue exhibited higher levels of 93 altered metabolites compared with the normal lung (p ≤ 0.05).

PAH tissue exhibited a distinct metabolic signature in comparison to the control lung (CL), as shown in the overview of metabolite analysis (Fig 1, Table 1). Profiling of metabolic analysis illustrated that 376 small molecule metabolites and 91 biochemicals from the PAH lung were significantly up-regulated (n = 83) or down-regulated (n = 8) compared with respective metabolites from the normal lung samples (*P*-value <0.05; FDR 1.1%), suggesting that PAH tissues have a unique pattern of global metabolomic heterogeneity.

### Polyamines and intermediates were changed in PAH Arginine metabolism

**Fig 2:**
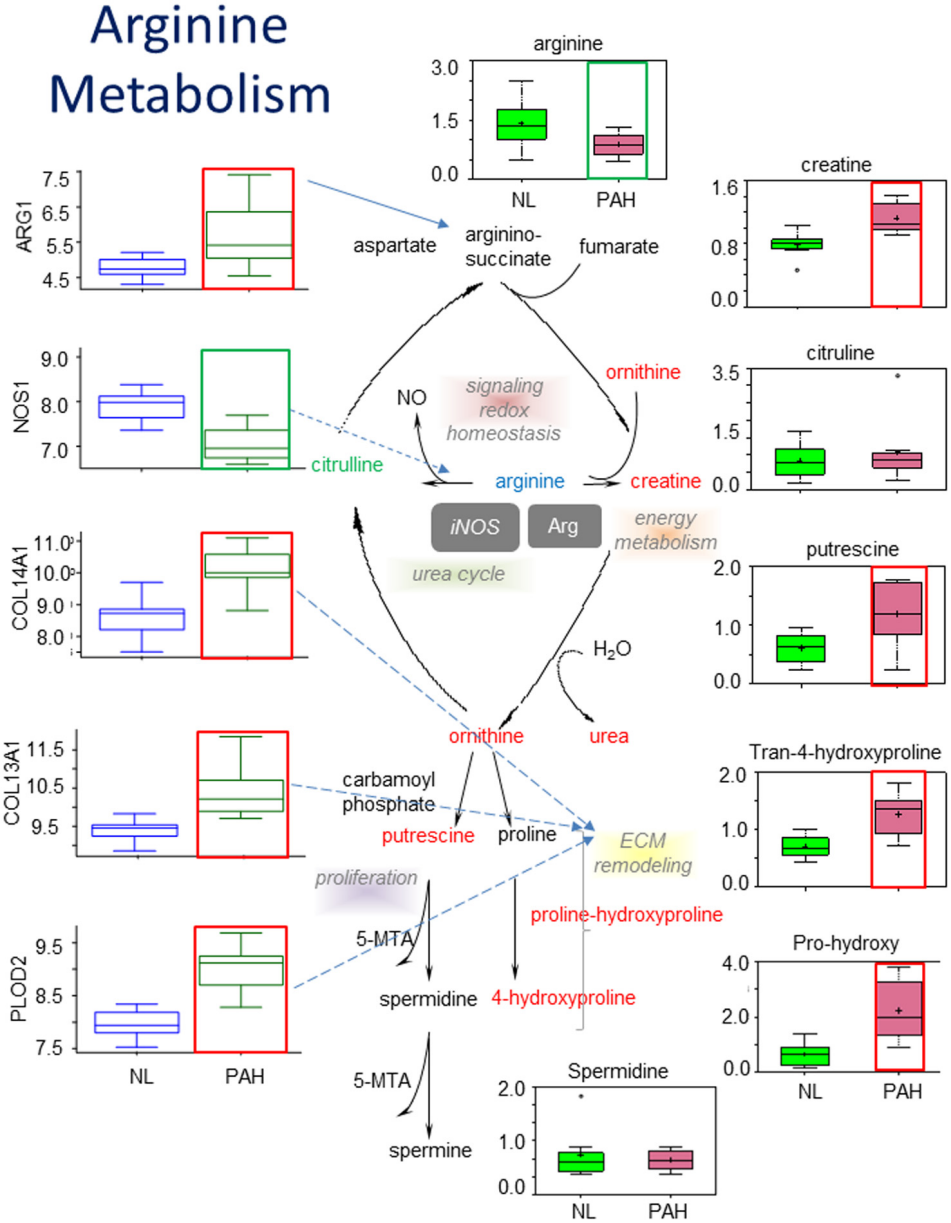
Polyamines and intermediates were changed in PAH Arginine metabolism. a) In all graphs, metabolic data for normal lungs are shown in filled green boxes for control, data for PAH lungs are represented in filled pink boxes, and the genes encoding key enzyme are shown in open blue boxes for control and open green boxes data for PAH lung (NL = control, PAH = pulmonary hypertension); P<0.05 for the group labeled with red frame. Quantities are in arbitrary units specific to the internal standards for each quantified metabolite and normalized to protein concentration (N = 8 for each box). PAH patient samples exhibited lower level of arginine and higher levels of creatine, putrescine, tran-4-hydroxyproline and pro-hydroxy. This metabolic disruption can contribute to the formation of advanced arginine end products and to the severity of PAH. The middle panel shows classical arginine pathways. The genes encoding arginase1 (ARG1), nitric oxide synthase 1, procollagen-lysine, 2-oxoglutarate 5-dioxygenase 2 (PLOD2), collagen type XIV alpha 1 (COL14A1) and collagen type III alpha 1 (COL3A1), were significantly changed in PAH lung compared with NL, as shown in left panel with colorless box (p<0.01).

We compared levels of polyamines and other substances involved in arginine metabolism between the PAH and normal lung tissue. Arginine is a key intermediate for extracellular matrix remodeling and for producing polyamines and nitric oxide (NO) in addition to protein synthesis. Polyamines are important in the stabilization of DNA, cell growth and differentiation. The metabolomic analysis shows that arginine was significantly decreased in PAH lung tissue compared to normal lung tissue (Fig 2), while other intermediates following arginine in the pathway were all significantly increased, including creatine, ornithine, and urea (Fig 2). These results suggest that in the pathological condition of PAH, the body uses arginine to synthesize other intermediates, such as NO, ornithine, and urea. In addition, the synthesis of polyamines and hydroxyl-proline was also increased.

In conjunction with our metabolomics study, we also performed molecular analysis. Microarray analysis showed that the gene encoding the enzyme arginase1 (ARG1) was significantly increased while nitric oxide synthase 1 (NOS1) was decreased in PAH. Both enzymes are involved in the homeostatic regulation of arginine and NO levels. We did not find significant changes of genes encoding iNOS (NOS2) and eNOS (NOS3) in PAH compared with controls. Increased expression of ARG1 combined with decreased NOS1 may be the result of a feedback mechanism due to disruption of the arginine metabolic pathway and excessive intracellular and extracellular NO levels. We also found that the PAH lung had significantly increased gene expression for genes involved in the biosynthesis of collagen, such as procollagen-lysine, 2-oxoglutarate 5-dioxygenase 2 (PLOD2), collagen type XIV alpha 1 (COL14A1) and collagen type III alpha 1 (COL3A1) (Fig 2)

### Enhanced Sphingolipids metabolism

**Fig 3:**
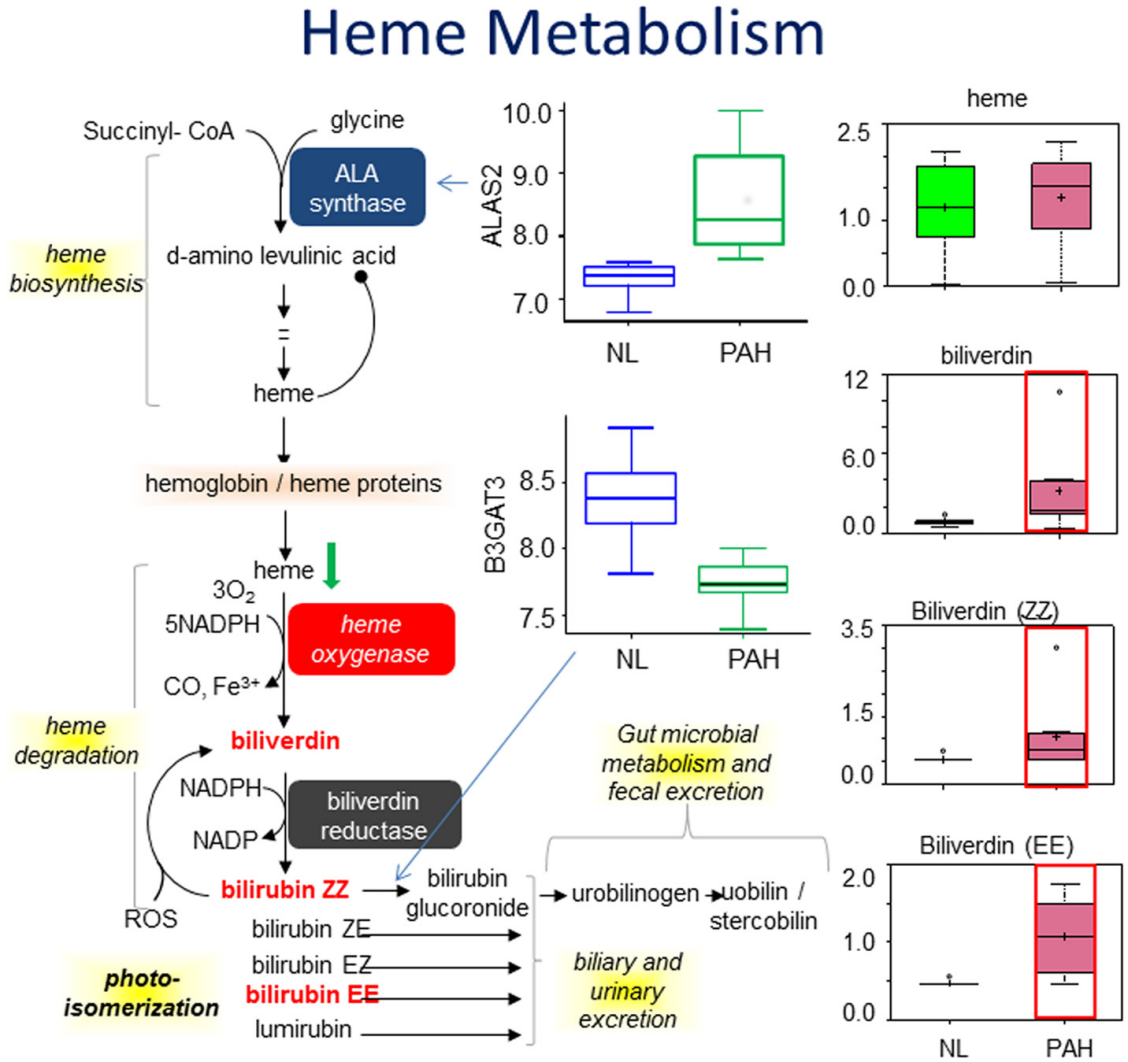
Increased heme degradation in heme metabolism. Heme is the most common porphyrin found in the body and complexes with cellular proteins to form hemoglobin, myoglobin, and cytochromes. This metabolite is initially synthesized from glycine and succinyl CoA and can be oxidized by heme oxygenase (HO) to generate bilirubin and the vasodilator carbon monoxide (CO). In PAH tissue, there was a significant accumulation of the biliverdin, biliverdin ZZ and biliverdin EE. Gene array analysis showed that the gene encoding aminolevulinate, delta-, synthase 2 (ALAS2) and a decrease in the gene coding beta-1,3-glucuronyltransferase 3 glucuronosyltransferase I (B3GAT3) were significantly increased in PAH (p<0.01). In all graphs, metabolic data for normal lungs are shown in filled green boxes for control, data for PAH lung are represented in filled pink boxes, and the genes encoding key enzyme are shown in open blue boxes for control and open green boxes data for PAH lung (NL = control, PAH = pulmonary hypertension). P<0.05 for the group labeled with red frame.

In sphingolipid metabolism, sphingolipid metabolites in PAH lung have variation with a scattered distribution pattern with a wider data range in PAH disease (Fig 3). Generally speaking, there was a trend of increasing metabolites of galactosylsphingosine, sphinganine, sphingosine and palmitoyl sphingomyelin in PAH (Fig 3). In comparison, the control lung tissue shows stable levels of all sphingolipids. To explore this finding further, we performed a gene array analysis and discovered significantly increased expression of genes encoding sphingolipid enzymes, including serine palmitoyltransferase, long chain base subunit 1(SPTLC1), serine palmitoyltransferase, long chain base subunit 3 (SPTLC3), neutral sphingomyelinase activation associated factor (NSMAF), sphingomyelin synthase 2 (sgms2), N-acylsphingosine amidohydrolase (non-lysosomal ceramidase) 2 (ASAH2) (Fig 3). Expression of sphingomyelin synthase 2 and neutral sphingomyelinase activation-associated factor was also increased, with a positive correlation to higher levels of sphingomyelin observed in PAH.

### Increased heme degradation in heme metabolism

**Fig 4:**
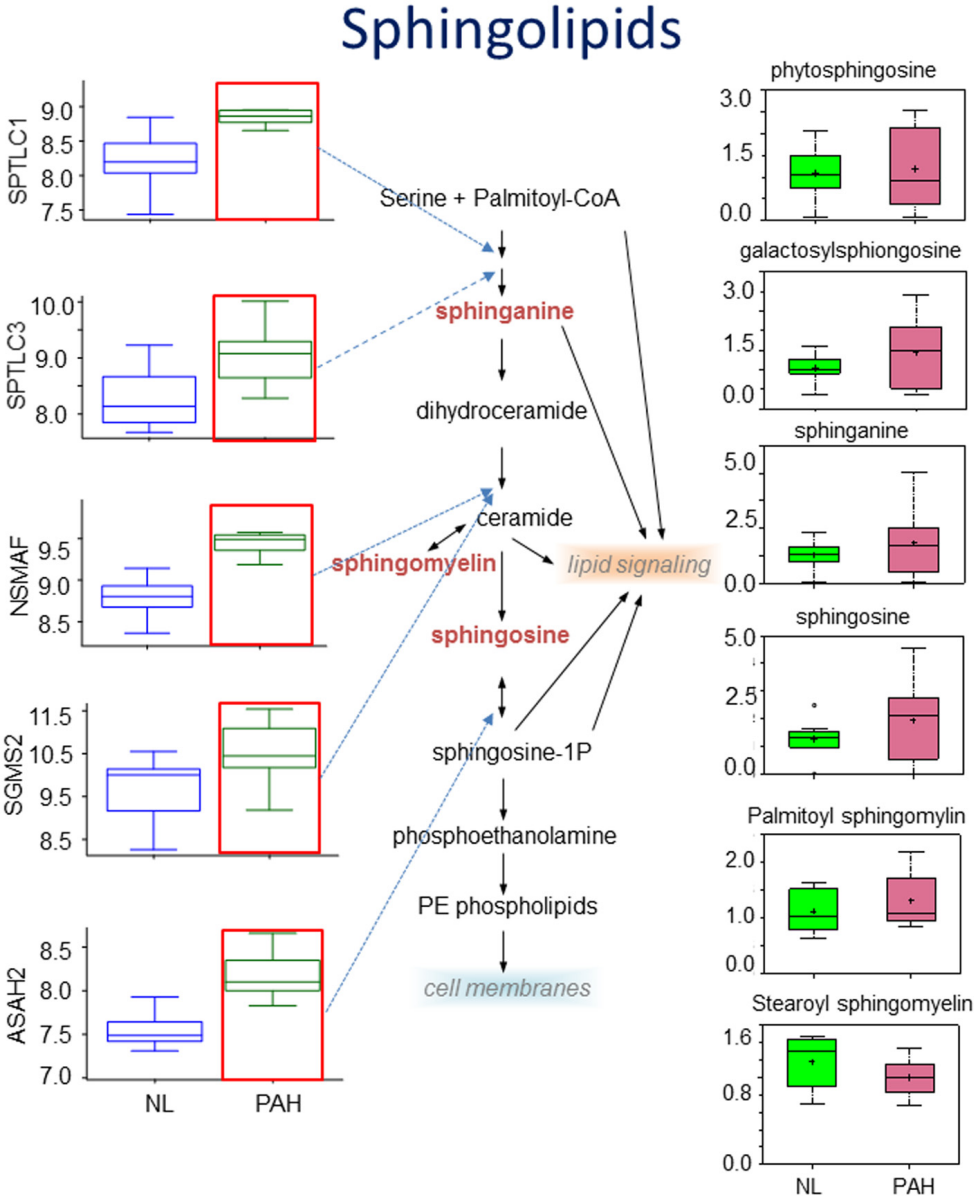
Enhanced Sphingolipids metabolism. a) Increasing metabolites of galactosylsphingosine, sphinganine, sphingosine and palmitoyl sphingomyelin were found in PAH. Gene array shows that expression of genes coding for the key enzymes that catalyze particular steps in the pathway are significant increased, including serine palmitoyltransferase, long chain base subunit 1(SPTLC1), serine palmitoyltransferase, long chain base subunit 3 (SPTLC3), neutral sphingomyelinase activation associated factor (NSMAF), sphingomyelin synthase 2 (sgms2), N-acylsphingosine amidohydrolase (non-lysosomal ceramidase) 2 (ASAH2) (P<0.01). In all graphs, metabolic data for normal lung are shown in filled green boxes for control, data for PAH lung are represented in filled pink boxes, and the genes encoding key enzyme are shown in open blue boxes for control and open green boxes data for PAH lung (NL = control, PAH = pulmonary hypertension). P<0.05 for the group labeled with red frame.

Heme is synthesized from succinyl-CoA and glycine, and then degraded into bilirubin through biliverdin. Biliverdin is oxidized from heme by heme oxygenase. The byproduct of heme degradation is a vasodilator, CO. Bilirubin (ZZ) and bilirubin (EE), are two of the products from biliverdin that contribute to the yellow color of bruises and urine, the brown color of feces, and the yellow discoloration in jaundice. We found a significant difference in heme metabolism in PAH samples compared to the normal control. Biliverdin, bilirubin (ZZ), and bilirubin (EE) were found significantly increased in PAH (Fig 4), which is in agreement with previous findings [11]. An increase of biliverdin and bilirubin indicate increased heme degradation, which may imply increased hemolysis in PAH. Furthermore, our gene array analysis found a significant increase in the gene encoding aminolevulinate delta-, synthase 2 (ALAS2) and a decrease in the gene coding beta-1,3-glucuronyltransferase 3 glucuronosyltransferase I (B3GAT3) for glycosaminoglycan biosynthesis (Fig 4). In PAH, the body cannot efficiently distribute oxygen throughout the body. In turn, it may increase heme synthesis by increasing the expression of ALAS2 and decreasing expression of B3GAT3. Decreased B3GAT3 may be due to negative feedback mechanisms from the accumulation of bilirubin in heme metabolism.

## Discussion

PAH is characterized by increased resistance in the pulmonary vasculature, eventually leading to right heart failure. We aimed to investigate alterations in several metabolic pathways that may contribute to the pathogenesis of PAH. Genetic profiling of enzymes involved in several metabolic pathways showed disrupted arginine metabolism, increased heme degradation and enhanced sphingolipid metabolic pathways in the human PAH lung, indicating that metabolic disruptions play an important role in the progression of PAH in its severe stages.

Polyamines are essential for cell proliferation and cell growth; increased levels of polyamines are primarily driven by an increase in polyamine enzymes, such as ornithine decarboxylase (ODC), polyamine transport or AdoMetDC [12]. In our results, levels of polyamines, such as putrescine, spermidine, and spermine were elevated. In addition, arginine and arginase activities were also found to be involved in polyamine regulation. Polyamines are required for nucleotide/DNA synthesis, and upregulation of the polyamine pathway could explain the known hyperproliferative phenotype of multiple cell types in the PAH lung.

Nitric oxide (NO) is the predominant signaling molecule for vasodilation [13,14]. NO dilates vessels by producing cyclic guanosine monophosphate (cGMP), which controls smooth muscle cell proliferation and migration, platelet aggregation, and leucocyte adhesion to the endothelium. There are two pathways involved in the production of NO: the NOS/NO pathway and the nitrate-nitrite-NO pathway. In a previous study, Zuckerbraun et al. suggested that increased NO levels may be the result of the PAH lung adapting to disease conditions [13]. In the body, arginase activity competes with NO production. Both ODC and AdoMetDC, the downstream products of arginase, are capable of restraining NO levels [15–17]. Additionally, NO functions to inhibit polyamine synthesis. Our study showed that a decrease of arginine was followed by significant increases of several other intermediates, including creatine, ornithine, and urea. These data indicate that in response to decreased arginine levels, there were increased polyamines and hydroxyproline synthesis. In conjunction with our metabolomic study, our gene array analysis showed that the gene encoding Arginase 1 (ARG1) was significantly increased while nitric oxide synthase 1 (NOS1) was decreased in PAH. Both ARG1 and NOS1 are key enzymes in the homeostatic regulation of arginine/ NO levels. Increased levels of ARG1 along with deceased NOS1 at the genetic levels may be the result of feedback mechanisms due to disrupted arginine metabolism and excessive intracellular and extracellular NO levels.

Studies have found that excess unbound NO will react with reactive oxygen species to produce peroxynitrite [18], a very reactive compound that further exacerbates cellular damage. Tyrosine residues reacts with peroxynitrite to produce nitrotyrosine, causing additional damage to airway epithelial cells [19] as well as pulmonary endothelial cells, which results in pulmonary vascular remodeling [18]. Our results of decreased arginine suggest the body’s possible reaction to hypoxic conditions in PAH, which correlates with disease progression into a more severe stage. But changes of the polyamine pathway could also be a consequence of the therapies that PAH patients were undergoing at the time of tissue acquisition. There are two separate classes of PAH therapies, the PDE5 inhibitors and the single FDA approved soluble guanylate cyclase activator (riociguat) are available in clinical treatment and both target the NO pathway directly. The possible effect of PAH therapies on the NO pathway need to be further studied. In PAH, NO signaling protects the vessel by helping the vessel adapt to new hypoxic conditions. However, as the disease progresses in severity, the NO signaling pathway becomes dysregulated. The nitrite anion, which provides NO to the arginine/NOS pathway, will also be dysregulated in order to adapt to PAH [13].

Our metabolic results show there is a low ratio of arginine-to-ornithine levels in the severity of pulmonary hypertension. The increased arginase activity decreases the arginine-to-ornithine ratio, and high arginase activity is linked with progression of pulmonary hypertension [20]. Increased arginase expression results in increased conversion of arginine to ornithine and urea, depleting the arginine pool available for nitric oxide synthesis. In addition, an increase of ornithine further depresses arginine levels. This cycle continues, widening the difference between arginine and ornithine. In response to low levels of arginine, arginase activity may increase as a result of a feedback mechanism. Indeed, we found that there was higher gene expression of ARG1, which encodes arginase in the PAH lung. Increased arginase activity may be the driving force for the decreased ratio of arginine to ornithine and citrulline. One of weakness of our study is that the metabolomics results come from the whole lung; therefore it may difficult to comment on the vascular specific outcomes. It would be better to verify the discovery metabolomics approach and profiling with a more targeted pulmonary vascular specific assay. Further experimentation using isolated pulmonary vascular tissue will help the transcriptomic/proteomic analysis of the pathway, which may support getting definitive statements. Several publications have disclosed the importance of arginine/ NO pathway by animal experimental studies [12, 15–19]. However, there is no biochemical data available that systemically shows metabolites changes of arginine/ NO pathway in human PAH lung, while, we have shown all the changes of the arginine and related metabolites in the PH lung. Furthermore, we have shown the correlation between the arginine pathway and collagen synthesis i.e. arginine-ornithine-4-hyrdoxyproline/proline-hyrdoxyproline-ECM pathway at both the metabolite and genetic level disclosing the genes that encode enzymes related to the collagen synthesis pathway.

Studies have shown that collagen synthesis is connected with hypoxia-induced pulmonary hypertension (HPH), and reducing collagen synthesis can limit the progression of HPH towards more severe stages, as well as limiting right ventricular (RV) hypertrophy [21–23]. PAH-related pulmonary vascular remodeling increases RV afterload, which in turn causes RV remodeling [24]. Hydroxyproline is one of the main components of collagen [17,25] and can promote collagen stability [26]. In our results, hydroxylated products were increased significantly. Hence, we speculate that elevated proline-hydroxyproline contributes towards pulmonary vascular remodeling by mediating collagen accumulation. In addition to vascular remodeling, collagen also supports the extracellular matrix (ECM)[27]. Thus, increased collagen levels also promote ECM remodeling.

Sphingolipids are a class of lipids that have sphingoid bases, and play a key role in maintaining the cell membrane, signal transmission and cell recognition. They are synthesized from serine and palmitoyl-CoA through several steps. It has been shown that alterations in expression of genes involved in sphingolipid metabolism could result in the progression of severe diseases, such as Alzheimer’s disease [28], which play a key role in promoting apoptosis through the generation of proapoptotic molecule, ceramide [29–31]. Sphingolipids also initiate the process of inflammation and insulin resistance in the pathogenesis of Type 2 Diabetes [32] and impaired sphingolipid homeostasis can result in the development of cancers [33,34]. Our results show increased levels of intermediates in this pathway such as sphinganine, sphingomyelin, and sphingosine, which suggesting that sphingolipid metabolism plays an important role in PAH disease. In conjunction with our experimental results, recent animal study from a hypoxic induced PAH model shows the importance of sphingolipid metabolism in PAH disease [35]. However, our data is based on biochemical analysis from human PAH lung, which is more realistic data. SphK1/S1P axis plays a significant role in the development of pulmonary arterial hypertension (PAH) by promoting PASMC proliferation, which is a major contributor to pulmonary vascular remodeling. Interestingly, our results showed that the change of intermediates are supported by significant changes in the genes encoding all enzymes in the sphinganine and sphingosine-related pathways. Indeed, the significant increase of SPTLC1 and SPTLC3, which encode serine palmitoyltransferase, were consistent with increased sphinganine. Significantly increased ASAH2, which encodes ceramidase, was consistent with increased sphingosine. SGMS2, the gene encoding sphingomyelin synthesis, was significantly increased, which was consistent with increased sphingomyelin. The increasing trend of sphinganine and sphingosine levels may be a compensatory mechanism in PAH to promote PASMC proliferation. In addition to its role in the vascular remodeling in PAH, increased sphingolipids may also play a compensatory role to enhance pulmonary vascular junction [36] in response to significantly increased pulmonary pressure in PAH. Accumulation of sphinganine in the vascular junction may use to repair and enhance the pulmonary vascular junction [37]. This hypothesis needs to be verified by further experimentation.

Heme metabolism consists of two parts: heme synthesis and heme degradation. Heme is synthesized from glycine and succinyl-CoA, and is a part of hemoglobin, myoglobin and cytochromes. Heme degradation converts heme into bilirubin with the help of heme oxygenase. Our results indicate that there was no significant change in heme synthesis, but heme degradation was significantly increased. Our results are in agreement with previous studies showing that serum bilirubin levels were significantly elevated in PAH and could serve as a predictor of PAH mortality [11,38]. The serum bilirubin levels were related to right atrial pressure and tricuspid regurgitation. Our gene and enzyme analysis showed significant changes in the levels of enzymes heme oxygenase and the genes ALA synthase 2 (ALAS2) and B3GAT3. To conclude, the alteration of heme metabolism in PAH may correlate with genetic changes.

Heme degradation is correlated with intravascular hemolysis, as lysis of red blood cells releases free hemoglobin, which further disassociates into free heme. Subsequently, heme serves as the source for the heme degradation pathway. Hence increased heme degradation may be the result of increased hemolysis, and may explain why heme biosynthesis was not increased in our study. Studies from Fox et al indicate that high erythrocyte creatine levels are an indicator of hemolysis [39]. In addition, Hebbe et al concluded that red blood cell survival is the gold-standard test for hemolysis [39]. They found that patients with PAH had subclinical hemolysis, as shown by higher EC levels [39]. In addition, elevated EC levels may also explain why creatine levels were increased in arginine metabolism. It has been claimed that the PH disease can be promoted by hemolysis through consuming NO [40]. Our patient’s data (see Table 1) show that mvO2, which provides an assessment of tissue oxygenation, shows an average 59%, which indicates that all patients are under hypoxic condition and would have increased heme metabolism related to hypoxemia. It would be interesting to see the dynamic changes in heme metabolism based on the severity of hypoxemia.

In heme metabolism, (HO)-1 helps protect the lung from oxidative stress and inflammation [41]. Specifically, HO-1 promotes heme degradation, which leads to antioxidant, antiapoptotic, antiproliferative, vasodilatory, and anti-inflammatory effects. Unlike other traits found in humans, the anti-inflammatory effect was found specifically in rats [42]. Biliverdin-Ixα, iron, and CO are products of heme degradation. The bilirubin-IXα transferred by Biliverdin-IX α helps to prevent inflammation. Iron can prevent the cell from undergoing oxidation and apoptosis. CO has a similar role to NO in preventing cell proliferation. As a result, a decrease of (HO)-1 caused by HO-1 promoter polymorphisms reduces heme degradation which can lead to many pulmonary diseases.

Limitation: we have analyzed the metabolic changes in PAH patients who are in the severe stage of PAH and require an organ transplantation. By analyzing end-stage PAH lung tissue, it is difficult to make conclusions about mechanisms that are pathogenic (i.e., causative) versus response to disease (i.e., compensatory). In addition, because these are patients who were scheduled to undergo transplantation, it is impossible to distinguish whether the metabolic changes were a consequence of disease development or a drug effect- as the patients surely were on multiple PAH-specific therapies, including prostanoids.

## Conclusions

In summary, our metabolomic profiles showed a disrupted arginine pathway, with increased NO, decreased arginine, increased S1P metabolites, and heme metabolites in severe human PAH lungs. The results suggest that PAH has specific metabolic pathways contributing to the vascular remodeling process in the advanced stage of pulmonary hypertension. Profiling metabolomic changes may provide a new understanding of pathogenic mechanisms involved in PAH and will aid in therapeutic targeting to specific metabolic pathways in the progression of PAH.
